# Role of T Follicular Helper Cells in Viral Infections and Vaccine Design

**DOI:** 10.3390/cells14070508

**Published:** 2025-03-29

**Authors:** Sohrab Ahmadivand, Robert Fux, Dušan Palić

**Affiliations:** 1 Faculty of Veterinary Medicine, Ludwig-Maximilians-University Munich, 80539 Munich, Germany; 2 Institute for Infectious Diseases and Zoonoses, Ludwig-Maximilians-University Munich, 80539 Munich, Germany; robert.fux@lmu.de

**Keywords:** viral infections, Tfh cells, mRNA vaccine, ferritin, germinal centers, B cells

## Abstract

T follicular helper (Tfh) cells are a specialized subset of CD4+ T lymphocytes that are essential for the development of long-lasting humoral immunity. Tfh cells facilitate B lymphocyte maturation, promote germinal center formation, and drive high-affinity antibody production. Our current knowledge of Tfh interactions with the humoral immune system effectors suggests that they have a critical role in supporting the immune response against viral infections. This review discusses the mechanisms through which Tfh cells influence anti-viral immunity, highlighting their interactions with B cells and their impact on antibody quality and quantity. We explore the role of Tfh cells in viral infections and examine how vaccine design can be improved to enhance Tfh cell responses. Innovative vaccine platforms, such as mRNA vaccines and self-assembling protein nanoplatforms (SAPNs), are promising strategies to enhance Tfh cell activation. Their integration and synergistic combination could further enhance immunity and Tfh responses (SAPN-RNA vaccines). In summary, we provide a comprehensive overview of the current insights into Tfh cells’ role during viral infections, emphasizing their potential as strategic targets for innovative vaccine development.

## 1. Introduction

Viral infections remain a global health challenge in human beings and animals due to their ability to evade immune responses, establish persistence, and undergo rapid mutation [1,2]. A robust immune response is the crucial element in countering viral infections, with T follicular helper (Tfh) cells playing a pivotal role in the generation of long-lasting immunity [3]. Tfh cells, a specialized subset of CD4+ T cells, are central to humoral immunity as they support B cell activation and promote germinal center (GC) formation and high-affinity antibody production. This process is primarily driven by CD40L-CD40 interactions and cytokines, particularly IL-21 [4]. Their role is especially crucial during viral infections, when they are essential for generating neutralizing antibodies [5,6,7]. In SARS-CoV-2 infections, a robust Tfh cell response is vital for protective immunity [7]. While Tfh cells promote rapid GC formation and antibody production in acute infections like influenza, their dysfunction in chronic infections such as HIV can impair immune responses and contribute to viral persistence [5,6].

The induction of Tfh cells can be influenced by factors such as species differences [8], individual characteristics [9], vaccine formulation (including adjuvants) [10,11], administration route and dose [12], and, most importantly, vaccine type and design [13], all of which affect antigen presentation and immune activation, highlighting the challenges in developing effective vaccines against viruses.

Recent advances in vaccinology have begun to leverage the unique capabilities of Tfh cells to improve vaccine efficacy. Novel platforms such as mRNA vaccines and self-assembling protein nanocages have demonstrated significant potential to enhance Tfh responses, driving robust GC reactions and high-affinity antibody production [14,15]. mRNA vaccines against SARS-CoV-2 are an example, where their lipid nanoparticle (LNP) formulation possesses intrinsic adjuvant activity and can mediate the induction of strong Tfh cell and GC responses [16,17]. Self-assembling protein nanocages, such as ferritin, offer advantages by mimicking the size and structure of viral particles and enhancing antigen presentation and Tfh cell activation through multivalent antigen display [18,19].

Such approaches and their synergistic combination to vaccine design that involve formulations with or without adjuvants targeting Tfh cells have shown promise in enhancing immune responses against several animal and human viruses [20,21,22]. Moreover, they offer cross-protection against different viral strains, making them a strong candidate in explorations of a “broad-spectrum” immunity approach [10,23]. The use of adjuvants, such as TLR and STING agonists has emerged as another critical strategy for optimizing vaccine-induced Tfh responses by promoting Tfh differentiation and GC reactions through dendritic cell activation, cytokine modulation, and enhanced antigen presentation [16].

This review provides a comprehensive overview of the current understanding of Tfh cells’ role in immune responses against viral infections across different species, emphasizing their interactions with B cells and significance in vaccine design. We address the potential for emerging strategies and technologies such as mRNA and self-assembling protein nanocage vaccine platforms and their synergistic combination, as well as potential adjuvants, to enhance Tfh cell responses, thereby offering insight into the development of next-generation vaccines for both human and veterinary applications.

## 2. T Follicular Helper Cells

### 2.1. Tfh Cell Characteristics and Function

**Figure 1:**
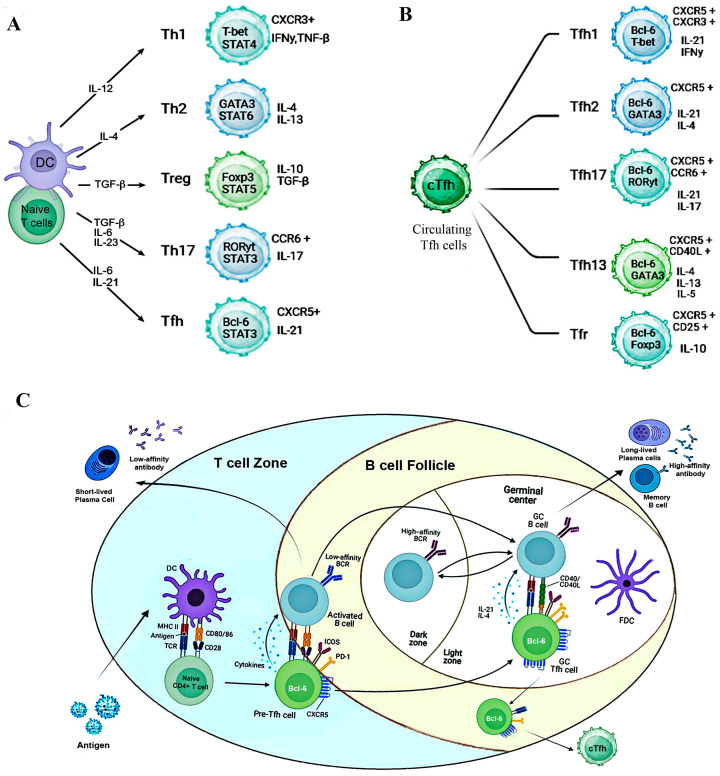
T follicular helper (Tfh) cell differentiation, subsets, and germinal center function. (**A**) Differentiation of T helper (Th) cell subsets. Interaction between dendritic cells (DCs) and naïve CD4^+^ T cells leads to polarization into Th1, Th2, and Th17 regulatory T cells (Tregs), and Tfh cells, driven by specific cytokines and transcription factors. (**B**) Subsets of circulating Tfh cells (cTfh). Tfh cells released into circulation (CD4^+^CXCR5^+^) are associated with distinct cytokine profiles and transcription factors such as T-bet, GATA3, RORγt, and Foxp3. (**C**) Germinal center (GC) Tfh differentiation. Naïve CD4^+^ T cells activated by DCs differentiate into pre-Tfh cells, which migrate to the T-B cell border and interact with activated B cells. In the GC, FDCs and Tfh cells (Bcl-6+, CXCR5+, PD-1+, ICOS+) promote B cell affinity maturation and differentiation into memory and long-lived plasma cells through costimulatory signals mediated by ICOS–ICOSL and CD40–CD40L interactions along with IL-4 and IL-21 cytokine secretion. Without Tfh interaction in GCs, B cells produce low-affinity antibodies as short-lived plasma cells. IL-6 is crucial for Tfh formation in animal models, but not in humans. Created with BioRender.com.

T lymphocytes, a crucial component of the adaptive immune system, play diverse roles in orchestrating immune responses against pathogens, including viruses. Among them, CD4^+^ T helper (Th) cells are essential in mediating the immune system defense by providing support to other immune cells, such as CD8^+^ cytotoxic T cells and B cells [24]. Upon activation, Th cells differentiate into various subsets, each tailored to combat specific types of pathogens (Figure 1A). These subsets include Th1, Th2, Th17, and the relatively more specialized Tfh cells, each with distinct roles in immune regulation. Th1 cells support cellular immunity and IgG1 and IgG3 antibody responses, while Th2 cells assist in humoral immunity, promoting IgE production [25]. Th17 cells are integral to mucosal immunity in areas like the lung and gut, and Tfh cells are uniquely involved in supporting humoral immunity and are essential for generating high-affinity antibodies [4]. The induced immune response can be suppressed by regulatory T cells (Tregs), another subset of CD4+ T cells [4].

Tfh cells, a specialized CD4^+^ T cell lineage, provide essential help to B-lymphocytes in GCs within secondary lymphoid organs (SLOs). They facilitate B cell affinity maturation through somatic hypermutation (SHM) and class-switch recombination (CSR), leading to the production of high-affinity antibodies [4,26]. This process also promotes B cell differentiation into memory B cells and antibody-secreting cells (ASCs), including plasma-blasts and plasma cells. Without interactions with Tfh cells and the GC reaction, B cells primarily produce low-affinity antibodies as short-lived plasma cells [27], (Figure 1C).

Tfh cells are characterized by the expression of the surface markers chemokine receptor 5 (CXCR5) [28], programmed cell death protein 1 (PD-1), [29], and inducible co-stimulatory molecule (ICOS), along with the transcription factor B cell lymphoma 6 (Bcl-6) [4,30]. Bcl-6 is regarded as the master regulator of the GC response, coordinating the interaction between Tfh and GC B cells, and it drives the transcriptional program that governs Tfh cell differentiation [31]. Tfh cells express CD40 Ligand (CD40L), which binds to CD40 on B cells in GCs, providing essential signals for B cell activation, survival, and differentiation into plasma cells and memory B cells [4].

They can also co-express T-bet (Th1), GATA3 (Th2), or RORγT (Th17), giving rise to distinct subsets: cTfh1, cTfh2, and cTfh17 cells found in the blood of both mice and humans and displaying a phenotype that closely resembles that of memory T cells (Figure 1B) [32]. The origin of circulating Tfh (cTfh) cells remains elusive, although their function as effective B cell helpers is well established [33]. cTfh cells, functioning alongside Tfh cells, can promote B cell differentiation into antibody-secreting cells [33]. cTfh cells are suggested to reflect GC reactivity, which was shown in both murine and human studies post-rituximab treatment, even in the absence of GC B cells [34]. However, further investigation is needed to fully define their role in GC responses.

In human blood, cTfh cells have been characterized by high CXCR5 and PD-1 expression, low to intermediate ICOS expression, and reduced Bcl-6 expression following Ebola vaccination, suggesting that continuous antigen stimulation may sustain or enhance Bcl-6 expression and influence cTfh cell function [31,35].

However, lymphoid tissue does not appear to contain equivalent Tfh subsets. In human blood, CD4^+^CXCR5^+^ cells are further divided into distinct cTfh subpopulations (cTfh1, cTfh2, and cTfh17) based on CCR6 and CXCR3 expression, each associated with specific transcription factors and cytokine secretion (Figure 1B) [33]. The functional diversity of Tfh cell subsets plays a crucial role in supporting the class switching of GC B cells, leading to the production of different immunoglobulin types. cTfh1 cells, characterized by a CXCR3+ CCR6− pattern, are primarily involved in antiviral immunity and responses to intracellular pathogens. They produce IFN-γ and IL-21, which drive IgG2a and IgG3 class switching in B cells [33]. In contrast, cTfh2 cells, which lack CXCR3 and CCR6, secrete IL-4 and IL-21, supporting immune responses to allergens and parasitic infections by promoting B cell class switching, especially to IgE [36]. cTfh17 cells, identified by their CXCR3+CCR6+ pattern, produce IL-21 and IL-17, and play a significant role in autoimmune responses and inflammatory conditions. IL-17 production enhances T–B cell interactions, promoting switching to IgG2a and IgG3 [37].

Follicular regulatory T (Tfr) cells play a vital role in maintaining immune tolerance and controlling autoimmunity by regulating GC responses and suppressing Tfh activity, primarily through the inhibition of Bcl-6 and promotion of Blimp-1 expression. Tfr cells express Treg markers like Foxp3 and co-express Bcl-6 but lack cytokines such as IL-4, IL-21, and CD40L, which are crucial for B cell activation. However, they have no or low expression of CD25 compared to Treg cells [36,38].

Tfh-like cells have also been identified in non-lymphoid tissues, which exhibit distinct marker expression patterns compared to Tfh cells in secondary lymphoid organs [36]. A newly identified subset, Tfh13, play a role in IgE production in allergic responses [39].

NKT cells can differentiate into CXCR5+PD-1+Bcl6+ follicular helper NKT (NKTfH) cells, which engage in CD1d-mediated cognate interactions with B cells, secrete IL-21, and support germinal center responses [40]. However, it has been shown that NKT cells do not acquire the NKTfH phenotype following viral infection [41].

Tfh cells are not characterized in many species due to the lack of antibodies for markers like CXCR5 and PD-1, which are commonly used in humans, mice, and pigs. Species-specific immune differences complicate comparisons [42], highlighting the need for further research on Tfh cells across species and diseases.

### 2.2. Tfh Cell Differentiation

Tfh cell differentiation is a multifaceted and highly regulated process that is essential for humoral immunity, enabling the formation of GCs and the production of high-affinity antibodies. Different stages of this process occur in distinct anatomical locations within SLOs and are influenced by specific molecular signals and cellular interactions [4,31].

Tfh cell differentiation begins with the activation of naïve CD4^+^ T cells by dendritic cells (DCs) in the T cell zone of SLOs. DCs present peptide–MHC class II complexes to T cells, triggering TCR signaling and co-stimulatory signals (e.g., B7 family: CD28-CD80/CD86). This initiates the upregulation of Bcl-6 and the differentiation into pre-Tfh cells [30]. Cytokines such as IL-6 (in animal models) and IL-21 (in both humans and animal models) activate STAT3, further enhancing Bcl-6 expression [43]. This, in turn, enables the expression of CXCR5, guiding pre-Tfh cells to migrate towards B cell follicles, where they encounter CXCL13-producing stromal cells and are poised for interaction with B cells [28].

At the T-B border, pre-Tfh cells interact with antigen-presenting B cells, reinforcing the Tfh transcriptional program. As Tfh cells mature in the germinal center, they adopt a CXCR5^+^, PD-1^+^, Bcl-6^+^ phenotype [44]. In the light zone, Tfh cells interact with B cells and follicular dendritic cells (FDCs), which present antigens to B cells, promoting survival, affinity maturation, and differentiation through CD40-CD40L and ICOS-ICOSL interactions [45]. Cytokines like IL-21 and IL-4 further support B cell maturation, with Tfh-derived IL-21 being essential for the formation and maintenance of germinal centers, as well as the development of long-lived plasma cells and memory B cells [4].

GC B cells return to the dark zone for SHM and CSR, with FDCs helping maintain the immune synapse. In the dark zone, centroblasts (activated B cells) undergo SHM and proliferation, driven by activation-induced cytidine deaminase (AID), [46].

Afterward, they migrate to the light zone, where they differentiate into centrocytes. This cycling between the dark and light zones ensures the selection of high-affinity B cells, while low-affinity or autoreactive B cells undergo apoptosis [44].

B cells also act as antigen-presenting cells (APCs) and produce cytokines like IL-6, which stabilize Tfh cell identity by promoting the expression of surface markers such as PD-1 and CXCR5. In turn, Tfh cells secrete IL-21, priming B cells for their roles in the germinal center reaction, including affinity maturation and differentiation into memory B cells and plasma cells [4,43]. IL-6 supports Tfh cell development in animal models, while IL-21 is essential for Tfh differentiation and function in both humans and animals [4].

Notably, recent evidence highlighted the role of BCR engagement in promoting GC B cell selection and differentiation into plasma blasts, despite the suppressed BCR signaling in GC B cells [47].

The differentiation of Tfh cell subsets is guided by the cytokine milieu and STAT activation [36,48]. Precursor Tfh (pre-Tfh) cells share developmental pathways with Th1, Th2, and Th17 cells. STAT1/STAT4 signaling (e.g., Type I IFN and IL-12) drives Tfh1 development, while IL-4/STAT6 promotes Tfh2 polarization but is inhibited by IL-6. Also, IL-6 and/or IL-21, and IL-23 activate STAT3 to drive Tfh17 differentiation, with RORγt aiding their specialization. ICOS signaling is also essential for Tfh17 differentiation. Most Tfr cells originate from Foxp3+ regulatory T cells (Tregs), although some can differentiate directly from naïve CD4+ T cells [48]. Tfr cells balance GC responses by producing IL-10 to support B cell growth and high-affinity antibodies while suppressing Tfh cells, GC B cells, and autoantibodies [48]. Recently, Tfh13 cells, characterized by IL-4, IL-5, and IL-13 co-production and the Th2 transcription factor GATA3, have been identified as key drivers of high-affinity IgE production [39,49].

The differentiation of Tfh cells is orchestrated by a network of transcription factors. Bcl6 plays a central role by repressing alternative CD4+ T cell fates, such as Th1, Th2, and Th17 cells, while enabling the expression of Tfh-associated genes [4]. In addition to Bcl-6, transcription factors like STAT3, TCF1, Ascl2, c-Maf, IRF4, and Batf synergize with Bcl-6 to promote Tfh cell differentiation and sustain the Tfh phenotype [31]. These factors coordinate the expression of key Tfh markers such as CXCR5, ICOS, and PD-1, facilitating Tfh cell migration to B cell follicles and interactions with B cells [31]. Conversely, negative regulators like Blimp-1 and STAT5 inhibit Tfh differentiation by promoting alternative effector T cell pathways, maintaining immune balance, and preventing excessive or inappropriate Tfh cell differentiation. These regulators also preserve immune homeostasis by preventing Tfh overactivation or differentiation into unwanted T cell subsets [36].

While the overall process of Tfh cell differentiation is conserved, the cytokine milieu varies across species. In mice, IL-6, IL-21, and Bcl6 are essential for Tfh formation, while in humans, Tfh generation relies on TGF-β, IL-12, IL-21, IL-23, and Activin A signaling, indicating the adaptability of Tfh cells to diverse immune environments [50].

Notable species-specific adaptations are also seen in non-mammalian vertebrates and farm animals. In pig lymph nodes, a CD4+ T cell population with an ICOS+Bcl-6+CD8α+ phenotype was identified, similar to human and murine germinal center Tfh cells. Blood-derived ICOShiCD25- and ICOSdimCD25dim CD4+ T cells induced the differentiation of CD21+IgM+ B cells into Ig-secreting plasma blasts. These cells were 3- to 7-fold enriched for Tfh-related transcripts (CD28, CD40LG, IL6R, and MAF) compared to naïve CD4+ T cells, as revealed by single-cell RNA sequencing [51]. Also, differences in lymphoid structures across species, such as the bursa of Fabricius in birds [52] or the absence of lymph nodes in fish [42], further hinder the characterization and functional study of Tfh cells that is needed to support tailored vaccine strategies.

## 3. Role of Tfh Cells in Viral Infections

Tfh cells, defined by CXCR5, ICOS, and PD-1 surface markers, along with the transcription factor Bcl-6 and the cytokine IL-21, are indispensable for mounting effective humoral immune responses during viral infections [4]. In acute infections, they drive antibody production, whereas in chronic infections, they contribute to immune regulation and viral persistence [5,6]. Their importance has been confirmed across species, including human and animals, against various viruses [5,6,7]. For instance, the IgG response to the vaccinia virus is diminished by 98% in the absence of Tfh cells [53].

Research in this context has predominantly focused on bulk GC-Tfh populations due to difficulties in isolating antigen-specific cells to study infections such as HIV and SIV infections, in which Tfh cells are associated with the development of broadly neutralizing antibodies, while Tbet+ GC-Tfh cells suggest a hybrid Tfh/Th1 function, though their precise roles require further investigation [54].

Circulating Tfh cells, which represent blood-resident counterparts of GC-Tfh cells, further underscore the importance of Tfh-mediated immunity in both contexts. Among the cTfh subset, Tfh1 cells, driven by IL-12 and STAT4, produce IFN-γ and enhance IgG2a and IgG3 antibody responses, which are critical in certain viral infections [37]. Longitudinal tracking of cTfh cells also showed activation of the cTfh1 subset and ASC expansion between 10 and 20 days post-infection, with the response magnitude correlating with the neutralizing- and total antibody titers [55].

IFN-γ from Tfh1 cells promotes the expression of T-bet in B cells, aiding in antibody isotype class switching and retention of B cells in the GC dark zone for affinity maturation during viral infections like lymphocytic choriomeningitis virus (LCMV) infections [56]. However, excessive IFN-γ can suppress GC responses, as was seen in some models of Plasmodium infection, indicating a fine balance in Tfh1-mediated regulation [57].

Tfh2 cells, characterized by IL-4 and IL-13 production, are involved in class switching to IgE and IgG1, which is particularly relevant in type 2 immune responses [36]. Although not traditionally associated with viral infections, sustained IL-4 production by Tfh2 cells can influence low-affinity antibody responses, which may contribute to viral immunity under certain conditions [56].

Tfh17 cells, producing IL-17 alongside IL-21, have been implicated in enhancing GC formation and supporting IgG2a/IgG3 switching. While their role in viral infections remains less well defined, their ability to stabilize T–B cell interactions in GCs could be significant in contexts requiring robust antibody production [37].

### 3.1. Tfh Cells in Acute Viral Infections

Acute viral infections rapidly induce Tfh responses, which are crucial for the production of virus-specific neutralizing antibodies. Mouse models of infections, such as LCMV, vesicular stomatitis virus (VSV), and influenza infections, have demonstrated the indispensable role of Tfh cells in protective humoral responses during acute infection. CD40L-deficient mice exhibit impaired GC formation, reduced memory B cell populations, and poor antiviral antibody production during LCMV and VSV infections [58]. Also, ICOS-deficient mice fail to generate robust GC responses during influenza infection, indicating the importance of ICOS signaling in Tfh functionality [59].

During acute viral infections, naïve CD4+ T cells differentiate into Tfh cells under the guidance of DCs and a cytokine-rich environment. Cytokines like IL-6, IL-21, and IL-27 activate STAT3 and induce the transcription factor Bcl-6, which drives Tfh lineage commitment while suppressing competing CD4+ T cell fates, including Th1, Th2, and Th17 cells [60].

Tfh differentiation is initiated through MHC-II/TCR interactions and stabilized by costimulatory signals such as ICOS and CD40L, which can be coordinated by Bcl-6, TCF-1, and mTORC2 [60]. Once differentiated, Tfh cells upregulate the chemokine receptor CXCR5, guiding them to B cell follicles. Within these follicles, primarily the IL-21-secreting Tfh cells ensure efficient GC formation and the generation of long-lived plasma cells and memory B cells. Notably, transient expression of T-bet in Tfh cells facilitates IFN-γ secretion, enhancing IgG2a class switching, especially during viral infections like influenza infections [4].

An acute Zika virus infection model in immunocompetent mice demonstrated that ZIKV elicits robust Th1-like Tfh cell and protective antibody responses. These Th1-like Tfh cells share phenotypic and transcriptomic features with both Tfh and Th1 cells but possess distinct markers and are T-bet dependent. They are essential for class switching to ZIKV-specific IgG2c antibodies and for sustaining long-term neutralizing-antibody responses, a function not performed by Th1 cells [61].

Early Tfh differentiation is linked to strong GC formation and high-affinity antibody production [56], but Tfh response kinetics are tightly regulated during acute viral infections. Early Tfh differentiation begins within 24–48 h, driven by high-affinity TCR interactions with DCs and sustained IL-6 and IL-21 signaling. IL-2 signaling, which supports Th1 differentiation, is suppressed by STAT5, promoting Tfh lineage commitment. Transcriptional regulators such as TCF-1 and Ascl2 also play key roles in early Tfh cell commitment during infections with viruses like influenza and LCMV [4].

During acute SARS-CoV-2 infection, activated cTfh cells (PD-1+ICOS+) with increased CD38 and reduced CCR7 expression peak within 14 days post-symptom onset. Spike-specific cTfh cells persist in convalescent individuals for at least 6 months, with a half-life of approximately 129 days, indicating their importance in long-term immunity [62]. The magnitude of Tfh responses is influenced by cytokines like IL-6 and/or IL-21 and the strength of the TCR signaling.

Moreover, this viral infection promotes spike-specific CXCR3+ Tfh cells, which are more persistent and better correlated with antibody responses, suggesting that prolonged infection can support the maintenance and recall of Tfh cells [7]. Notably, cTfh1 clones show a significant overlap with CXCR3+ tonsil-derived Tfh cells [16,63].

Similarly, strong Tfh generation during influenza infection depends on the localized recognition of antigens by CD4 effector cells and the presence of continuous infection-derived signals, reinforcing the idea that sustained infection is key to driving effective Tfh responses [64]. These findings indicate that the duration and persistence of the infection are essential for inducing durable Tfh responses, providing insights for improving vaccine efficacy by replicating the continuous antigenic stimulation observed in natural infections.

### 3.2. Tfh Cells in Chronic Viral Infections

Chronic infections with viruses such as HIV, hepatitis C virus (HCV), and chronic LCMV present unique challenges to Tfh cells’ functionality. Persistent antigenic stimulation during chronic infections skews CD4+ T cell differentiation toward the Tfh lineage, leading to increased Tfh cell frequencies. However, this expansion often comes at the cost of functionality, with chronic immune activation impairing Tfh cell support for B cells, disrupting GC dynamics, and resulting in delayed or suboptimal antibody responses [5].

In chronic LCMV infection, the loss of Th1 cells is accompanied by an increase in Tfh cells; however, these Tfh cells fail to sustain robust GC reactions, produce lower levels of IL-21, and delay neutralizing-antibody production, ultimately compromising the viral control [65]. During HCV infection, CXCR3+ cTfh cells are associated with stronger neutralizing-antibody responses, reflecting pathogen-specific influences on cTfh function [66]. During HIV infection, expanded Tfh populations contribute to polyclonal B cell activation, hypergammaglobulinemia, and defective antibody responses. Nonetheless, circulating PD-1+CXCR3–CXCR5+ memory Tfh cells remain highly functional and are associated with the development of broadly neutralizing HIV antibody responses [67]. In HIV infection, Tfh cells within GCs also evade CD8+ T cell-mediated killing and serve as latent viral reservoirs, complicating both immune clearance and therapeutic interventions [68]. Pre-Tfh cells in secondary lymphoid tissues, marked by high CCR5 expression, are particularly susceptible to this infection. These cells may transition into fully functional Tfh cells, upregulating PD-1 during chronic infections and further contributing to viral persistence [5].

Therapeutic strategies such as PD-1 blockade and recombinant IL-21 are being explored to enhance Tfh-B cell interactions and improve antibody responses in chronic infections. However, these approaches carry risks, including heightened T cell activation, which could trigger viral reactivation or promote the formation of new reservoirs within GCs [69].

Despite these challenges, Tfh cells remain crucial for vaccine efficacy against chronic viral infections. Enhancing Tfh responses through targeted therapies or vaccines could improve antibody generation to combat rapidly mutating viruses such as HIV and HCV. Understanding the transcriptional regulation of Tfh differentiation, including the roles of Bcl-6 and Blimp-1, could provide valuable insights for tailoring immune responses in chronic viral infections.

## 4. Targeting Tfh Cells Through Vaccine Design

### 4.1. Tfh Cells and Vaccines

Vaccines are effective in preventing viral infections, reducing transmission, and establishing herd immunity [70,71,72]. Humoral immunity, driven by high-affinity antibodies produced by B cells, is essential for vaccine efficacy [4]. Consequently, the efficient elicitation of Tfh cell responses to vaccine antigens has become a key area of research. Serological responses to influenza, yellow fever, malaria, and SARS-CoV-2 vaccines correlate with cTfh activation, particularly those expressing markers such as CXCR5+ and ICOS+, and the Th1-polarizing conditions in these infections generally result in the predominant generation of type 1 Tfh cells [55].

Vaccine type significantly affects Tfh cell activation and overall efficacy. Live attenuated vaccines induce the strongest Tfh responses, mimicking infection, while inactivated and subunit vaccines, though safer, often require adjuvants to enhance immunogenicity [17,73]. Aligned with this, Devarajan et al. [64] have reported that a second dose of a live attenuated influenza vaccine during the effector phase can effectively replicate infection-like conditions, eliciting robust Tfh responses.

Studies on inactivated vaccines have shown that Tfh responses are typically induced only when an adjuvant is included and after administering at least three doses [73]. For example, the BBV152 vaccine, a whole-virion inactivated SARS-CoV-2 formulation, achieved this effect by incorporating a TLR 7/8 agonist molecule (IMDG) adsorbed to alum [71]. However, these findings are further complicated by safety concerns, particularly adjuvant-dependent effects associated with inactivated vaccines [74].

Despite the role of the CpG domain in DNA vaccines, the Tfh response has been shown to be further enhanced by chitosan encapsulation of a plasmid encoding the VP1 protein of coxsackievirus B3 [75]. However, the commercialization of this type of vaccine is hindered due to concerns related to genetically modified organisms [76].

Modulating the GC Tfh abundance can influence B cell recruitment and epitope-specific antibody production [77], further indicating the importance of strong CD4+ T cell responses in vaccination [5,78]. Furthermore, GC-Tfh cells rely on continuous antigen presentation for their maintenance, and adjuvants that support CD4+ T cell priming or prolong GC-Tfh cell activity can improve humoral immunity [79]. Lymph node fine needle aspirates (FNAs) offer a minimally invasive approach to assessing GC-Tfh cells and GC biology, providing insights into Tfh and GC dynamics in human and primate models [80].

Nonetheless, studying Tfh responses and the exact role of Tfh subsets in blood is challenging, as GC-Tfh cells primarily reside in lymphoid tissues [4]. Recently activated cTfh cells (CXCR5+ICOS+, Ki67+PD1hi) can be identified and are distinct from the predominant resting memory cTfh cells [81,82]. Following influenza vaccination, the frequency of influenza-specific activated cTfh cells increases, correlating with the IgG responses [82].

The subunit SARS-CoV-2 RBD vaccine, composed of the RBD protein expressed in Drosophila S2 cells and formulated with an alum adjuvant, has elicited significant antigen-specific Tfh cell responses (predominantly Tfh1 and Tfh1-17 subset responses and strong GC responses) in the lymph nodes and spleen, playing a critical role in the induced humoral responses [16]. Additionally, the magnitude of the Tfh and GC responses is crucial for the immunogenicity of these vaccines, which can be directly influenced by the vaccine dose and frequency, i.e., boosters [83].

In the case of the SARS-CoV-2 vaccine, although ICOS+CXCR5+CD4+ T cells recognized the RBD antigen by day 7 post-vaccination, indicating rapid Tfh cell activation, booster vaccination—known to modulate the magnitude of Tfh and GC responses—further enhanced Tfh activation by preferentially increasing the CXCR3+ (Tfh1 and Tfh1-17) subset over the CXCR3− (Tfh2 and Tfh17) subset in the spleen and lymph nodes [17]. A nonadjuvanted trivalent influenza vaccine increased the CXCR3+ cell frequency [66], while Tfh17 cells were induced during the rVSV-ZEBOV Ebola vaccine response [35]. Both Tfh1 and Tfh17 responses were observed in SARS-CoV-2 mRNA-LNP vaccines [84]. Additionally, Yin et al. [85] reported a shift from cTfh2 and cTfh17 to cTfh1 in low responders to the hepatitis B vaccine, further highlighting the importance of studying Tfh subsets.

Studies on influenza vaccines have shown that they primarily activate memory B cells (MBCs), enhancing antibody responses [33,86]. Recombinant RBD protein induces Tfh cells, which effectively promotes MBC differentiation into antibody-secreting cells, unlike non-Tfh cells. Elevated IgG and IL-21 levels in Tfh-MBC co-culture supernatants after RBD stimulation suggest that Tfh cells amplify the memory B cell response. This role of Tfh cells in boosting antibody production has also been observed with other vaccines, such as papillomavirus vaccines [86].

Moreover, some vaccine platforms (such as mRNA) promote a shift from IL-4 to IFN-γ production in Tfh cells, which is associated with improved immune responses [87]. This suggests that the ability to elicit functional IFN-γ-producing Tfh cells could be also a key factor in improving vaccine efficacy.

Other innovative strategies, such as incorporating SAPNs into mRNA vaccines [88] and utilizing adjuvants, have shown promising potential in enhancing Tfh cell activation, warranting dedicated discussion to explore their mechanisms and applications in enhancing vaccine efficacy for both human and animal viruses.

### 4.2. RNA Vaccines: Immunity and Tfh Cell Responses in Humans and Animals

**Figure 2:**
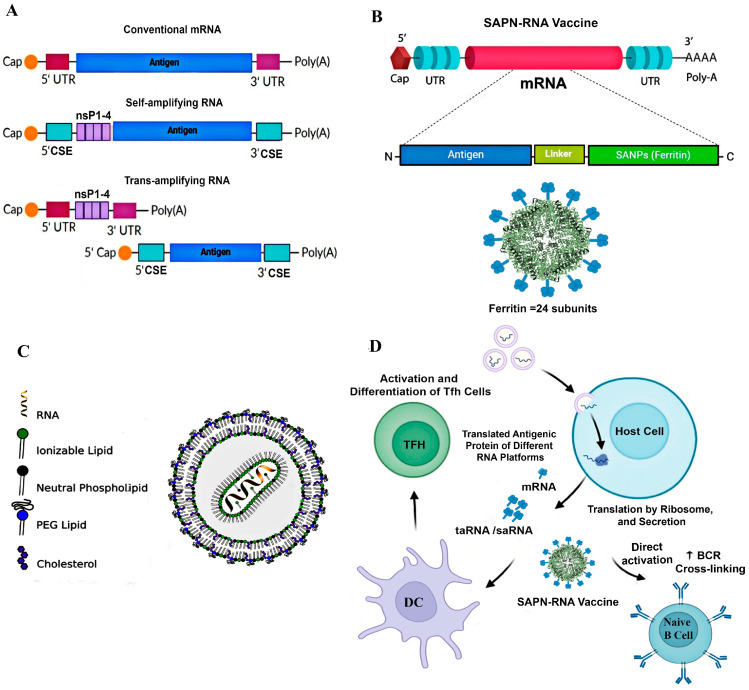
Design and antigen expression mechanism of various mRNA vaccine platforms. (**A**) Comparison of conventional mRNA, self-amplifying RNA (saRNA), and trans-amplifying RNA (taRNA) vaccine designs. (**B**) SAPN-RNA vaccine design: Integration of self-assembling protein nanoparticles (e.g., ferritin with 24 subunits) in mRNA vaccines for multivalent antigen display. (**C**) Key components of lipid nanoparticles (LNPs). (**D**) Mechanism of action of mRNA vaccines: SAPN-RNA vaccine directly activates naïve B cells and is efficiently processed by antigen-presenting cells, such as dendritic cells (DCs), facilitating Tfh cell activation and differentiation from CD4+ T cells by engaging PRRs, while other formulations mainly rely on LNP adjutant properties. This along with promoting multiple BCR cross-linking and its slow-release properties ensure robust, durable, and stable antibody responses at a lower dose. Cap: m7G; UTR: untranslated region; CSE: conserved sequence element; nsP1–4: alphavirus nonstructural proteins 1–4. Created with BioRender.com.

RNA vaccines, such as messenger RNA (mRNA) vaccines, have emerged as a transformative technology in vaccinology, offering rapid development and adaptability to various pathogens (Figure 2). Their scalability was notably demonstrated during the SARS-CoV-2 pandemic [89]. At the core of conventional mRNA vaccine development is the molecular design of synthetic mRNA, which incorporates critical elements to optimize stability, prevent degradation, and ensure efficient translation. Conventional mRNA vaccines are transcribed in vitro from DNA templates, with stabilization achieved through 5′ capping, untranslated regions (UTRs) and poly-A tails to enhance translation and reduce degradation [90] (Figure 2A).

In addition to their success in combating SARS-CoV-2, mRNA vaccines are being explored for other viral infections and even cancer therapies. In this context, self-amplifying RNA (saRNA) platforms have emerged, offering enhanced vaccine efficiency by amplifying antigen production within cells, reducing the necessary mRNA doses, and lowering production costs [91,92]. In contrast to conventional mRNA vaccines that encode only the antigen of interest and rely entirely on the host cell machinery for translation [87], saRNA vaccines incorporate replication machinery derived from alphaviruses, including nonstructural proteins (nsP1–4), enabling intracellular RNA amplification [93] (Figure 2A). These vaccines utilize LNPs to deliver mRNA into host cells, which is then translated into antigen proteins that stimulate both humoral and cellular immune responses [94] (Figure 2C). In addition to LNPs, several alternative delivery systems under investigation include polymeric nanoparticles (PEI and chitosan) for stability, inorganic nanoparticles (gold and silica) for targeted delivery, and cell-penetrating peptides (CPPs) for direct mRNA entry into cells [95,96].

More recently, self-assembling protein nanoparticles (SAPNs) have emerged as an approach to stabilizing antigenic proteins in RNA vaccines and improve their presentation to the immune system, thereby amplifying the efficacy of the vaccines. We refer to this approach as the “SAPN-RNA vaccine” (Figure 2B). The immune responses and Tfh cell activation induced by both conventional mRNA and saRNA vaccines can be further enhanced through this incorporation [22,88] (Figure 2D).

mRNA vaccines have shown promising results in promoting adaptive immunity against various human and animal viruses. mRNA vaccines, such as Moderna mRNA-1273 and Pfizer/BioNTech BNT162b2, induce strong immune responses, including increases in memory CD4+ T cells, circulating Tfh cells, and cytotoxic T cells. Although antibody levels decrease over time, the stability of memory T and B cells supports long-term immunity. mRNA vaccines also generate superior neutralizing antibodies and have lower reactogenicity compared to inactivated and viral vector vaccines, offering enhanced protection against viral infections [13,97]. mRNA vaccines also induce both humoral and cellular immunity. Upon delivery, the mRNA is translated into antigens in the cytosol, leading to their degradation and presentation on MHC-I molecules, activating cytotoxic CD8+ T cells. Simultaneously, secreted or membrane-bound antigens are processed via MHC-II pathways, stimulating CD4+ T cells and promoting antibody production by B cells [98].

SARS-CoV-2 mRNA vaccines are an example of vaccines that promote strong Tfh cell activation and germinal center responses, leading to higher neutralizing-antibody production and the generation of long-lived plasma cells and memory B cells (Table 1). LNP formulations activate innate immune responses via pattern recognition receptors (PRRs), such as Toll-like receptors (TLR7 (mouse and human) and TLR8 (human) that detect the presence of foreign RNA, triggering the production of type I interferons and other inflammatory cytokines [98,99]. Through this process, the ionizable lipid in the LNPs triggers the production of cytokines, such as IL-6 in mice [17]. Notably, these vaccines promote a shift from IL-4 to IFN-γ production in Tfh cells, which is associated with improved immune responses [87].

In addition to SARS-CoV-2, mRNA vaccines for other viruses, including influenza and Zika, have been developed and tested in animal models and showed similar immune responses in terms of Tfh and GC activation. For example, mRNA vaccines targeting the influenza virus in mice have been shown to activate antigen-specific Tfh cells, leading to enhanced GC responses and high titers of neutralizing antibodies, a result supported by the secretion of IL-21 [94]. The induction of robust B cell responses following influenza mRNA vaccination is closely associated with the presence of circulating hemagglutinin-specific ICOS+ PD-1+ CXCR3+ Tfh cells [100]. Similarly, an mRNA vaccine developed against the Zika virus has demonstrated that vaccination increases Tfh responses, which play a crucial role in eliciting a sustained neutralizing-antibody response, providing lasting protection against Zika infection [101].

mRNA vaccines are also gaining attention in veterinary medicine as innovative solutions for managing viral infections in farm animals [102]. However, most animal studies, including those using animal models and veterinary species, focus on GC-Tfh cells, whereas the human data predominantly relate to cTfh cells, primarily due to sampling limitations. Recent research has highlighted their ability to induce robust immune responses, including the activation of Tfh cells, mirroring their success in human medicine [12], optimizing the mRNA-B sequence for expressing the rabies virus glycoprotein (RABV-G), and evaluating its immunogenicity across various animals. A single dose of the mRNA-B-LNP vaccine induced rapid and long-lasting protective antibody responses in mice, outperforming inactivated vaccines in dogs by generating higher neutralizing-antibody titers. Two doses provided sustained humoral responses, strong Tfh and GC B cell activity, and Th1-biased cellular immunity. Additionally, the vaccine remains stable as a liquid formulation at 2–8 °C for up to two months, showcasing its practical application in veterinary medicine. Studies on zoonotic Rift Valley fever virus (RVFV) mRNA vaccines targeting various viral proteins, including Gn-head, Gn-stem, Gc, and GnGc, revealed high frequencies of GC B cells and Tfh cells in BALB/c mice, as determined by flow cytometry of inguinal lymph node cells. These immune responses were also confirmed in rhesus macaques, demonstrating the vaccine’s potential for cross-species immune activation [103].

In livestock, mRNA vaccines have shown promising results against several viral diseases. These vaccines, such as those for Foot-and-Mouth Disease Virus (FMDV) [104], avian influenza [105], and Bovine Viral Diarrhea Virus (BVDV-1) [106], induce strong immune responses, including the production of virus-specific antibodies (IgY and IgA) and activation of CD4+ T cells and Tfh-like cells. These responses significantly reduce viral loads and pathology. For BVDV-1, both cap-dependent and cap-independent mRNA vaccines generate robust neutralizing-antibody titers in many species, including mice, guinea pigs, and goats. In swine, mRNA vaccines targeting African Swine Fever (ASF), and Porcine Reproductive and Respiratory Syndrome Virus (PRRSV) [107,108,109] have shown excellent safety and efficacy, boosted neutralizing-antibody production, and activated both CD4+ and CD8+ T cells with increased cytokine secretion. For PRRSV, GP5-mRNA vaccines also demonstrated superior immunogenicity compared to traditional vaccines [109].

**Table 1:** Tfh cell responses induced by mRNA and self-assembling protein nanocage (SAPN) vaccines against viral pathogens.

Vaccine Type	Virus(es) (Antigen)	Species	Adjuvant	Tfh-Related Immune Responses	Ref(s).
mRNA	SARS-CoV-2 (Spike Protein)	Human	-	Induces strong neutralizing-antibody response and persistent cTfh cells for six months, with potent GC B and Tfh responses, including increase in Tfh1 cells after the first dose	[15,110]
mRNA	HIV-1 (Env), ZIKV (prM-E); influenza virus (HA)	Mice and rhesus macaques	-	Potent T follicular helper and germinal center B cell responses	[94]
mRNA	RVFV (Gn and Gc)	Mice and rhesus macaques		Induces potent GC B and Tfh cell responses 10 days after booster	[103]
saRNA	SARS-CoV-2 (RBD-TM)	Mouse, NHP, and hamster models	Alum	Robustly activated Tfh cells; cross-reactive responses against heterologous variants and VOCs, lasting at least 12 months in NHPs (Cynomolgus macaques)	[93]
Ferritin–mRNA	HIV-1 (Env)	Mice	-	Induced specific GC B cells and memory B cells in spleen. Tfh cells and GC Tfh cells were also observed in vaccinated mice	[88]
Ferritin–mRNA	SARS-CoV-2 (RBD)	Mice	-	Elicited robust titers of specific antibodies, including neutralizing antibodies with increased expression of IFN-γ and IL-4	[111]
LuS–mRNA	Rotavirus (P2-VP8*)	Mice and guinea pigs	Alum	Induced the highest specific IgG titers compared to conventional mRNA and subunit vaccines, which remained constant over time	[112]
LuS–mRNA	*SARS-CoV-2 (RBD)*	Mice	*-*	Strong neutralizing-antibody responses and protection against the Delta variant	[113]
LuS–saRNA	*HIV (gp120)*	Mice	ISCOM-like	Elicited GC B and Tfh cell responses with a balanced IgG1/IgG2 humoral immune response	[22]
Ferritin	*Influenza virus* *(HA)*	Mice and pigtail macaques	AddaVax	High antibody titers and directly activated germinal centers through a B cell-intrinsic mechanism	[114]
Ferritin	*HBV (preS1 domain)*	Mice	CpG-1826	SIGNR1+ dendritic and macrophage cells activated Tfh and B cells, driving strong and lasting antibody responses	[16]
Ferritin	*SARS-CoV-2* (Spike Protein)	Mice	ALFQ/Alhydrogel	One dose of vaccine induced IL-21-producing spike-specific Tfh and GC B cells, S-2P-specific IgM/IgG, and robust cross-neutralizing antibodies by day 7	[10]
Encapsulin	Rotavirus (VP8*)	Mice	Alhydrogel	Induced specific IgG1, IgG2a, and neutralizing antibodies with superior immunogenicity over subunit vaccine	[115]
LuS and E2	RVFV(Gn)	Mice and lambs	TS6	Protection correlated with the induction of robust neutralizing antibodies in both species	[116]
sHSP	*Antigen ovalbumin (OVA)*	Mice	Alum	OVA-specific IgG1 was detectable by day 5, with strong mucosal sIgA and germinal center B and Tfh cell responses	[18]

In aquaculture, mRNA vaccines are emerging as a potential solution to viral diseases, which pose a significant challenge to the industry [117,118,119]. While promising for both fish [120] and crustaceans [121], mRNA vaccines face substantial challenges due to their distinct and relatively simple immune systems [42,122]. For example, fish lack germinal centers and instead rely on diffuse lymphoid tissues for B cell activation, with Tfh-like cells playing a role in supporting immune responses [42]. These findings highlight the potential of mRNA vaccines in veterinary medicine, though key aspects, such as Tfh cell responses, remain underexplored.

To further enhance immune responses and Tfh cell activation, mRNA and saRNA vaccines can be combined with molecules that activate immunostimulatory receptors, such as OX40, CD137, or CD40, or molecules that block immune checkpoints like PD-1, PD-L1, and CTLA-4. Such combinatory strategies have been shown to potentiate the efficacy of these vaccines. For instance, activating OX40 in combination with saRNA vaccines has been tested in COVID-19 models, resulting in enhanced T cell cytokine responses and improved viral clearance [123]. Similarly, combining IL-12-MOP with the BNT162b2 SARS-CoV-2 vaccine significantly boosted immune responses in animal models [124].

The SAPN-RNA approach, incorporating self-assembling protein nanocages to mRNA vaccines, offers a novel strategy to boost immunity and activate Tfh cell responses. This could allow mRNA vaccines to be more effective, potentially requiring lower doses and offering better stability, making it a promising strategy for both human and veterinary vaccines.

### 4.3. Self-Assembling Protein Nanocages (SAPNs) as Vaccine Platforms: Immunity and Tfh Cell Responses

**Figure 3:**
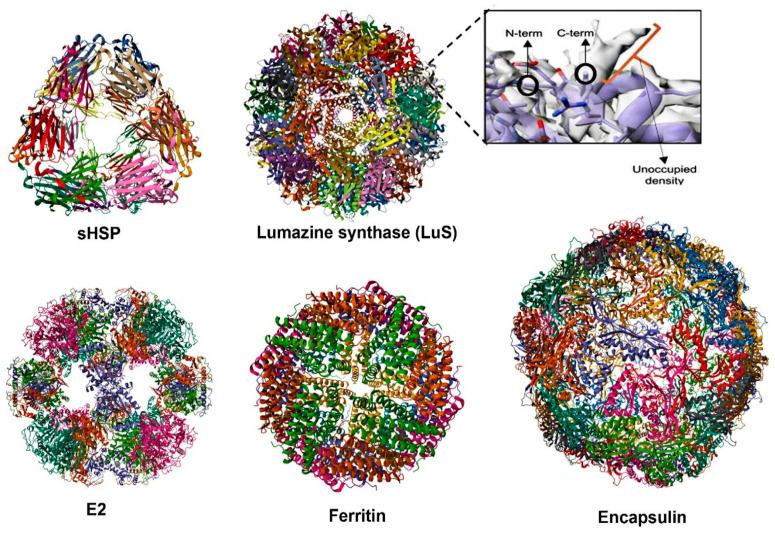
Schematic structures of natural self-assembling protein nanocages (SAPNs). sHSP: small heat shock protein; MjsHSP16.5 (~16.5 nm, 24-mer; PDB ID: 8WP9); lumazine synthase (~16–24 nm, 60-mer; PDB ID: 8F25) with exposed C- and N-termini on the surface; E2 Dihydrolipoyl Acetyltransferase (~24 nm, 60-mer; PDB ID: 1B5S), ferritin (12 nm, 24-mer; PDB ID: 3BVE); and Encapsulin (20–100 nm, 60-, 180-, or 240-mer; PDB ID: 3DKT). Created with BioRender.com.

Self-assembling protein nanocages (SAPNs) occur naturally in various organisms and have emerged as promising platforms for novel vaccine development (Figure 3). These nanoscale structures, ranging from 10 to 100 nm in size, closely mimic the size and shape of viruses, enabling the multivalent display of antigens in highly ordered architectures [19,21]. This design enhances their uptake and processing by antigen-presenting cells, directly activates naïve B cells, and facilitates efficient cross-linking of B cell receptors (BCRs) [19]. Like mRNA vaccines, they simulate innate immune responses through PRRs (TLR-4) engagement [125]. Moreover, SAPNs promote robust interactions with immune cells, such as Tfh cells, driving germinal center responses that are critical for generating durable and high-affinity antibody responses (Table 1).

SAPNs exhibit remarkable thermal and pH stability, monodispersity, a small and uniform size, biodegradability, biocompatibility, cost-effective mass production, pseudoreversible spontaneous assembly/disassembly, and the potential for surface conjugation [19,21,126].

Importantly, SAPNs can favor the co-delivery of antigen and adjuvant to the same immune cell, which enhances the adjuvant effects and limits off-target effects [127]. SAPNs provide versatile scaffolds capable of accommodating a range of antigens, including those from enveloped viruses via substituting lipid membranes and matrix proteins, and rescuing insoluble antigenic proteins for vaccine applications [126]. These properties address significant challenges in the stability and immunogenicity of subunit and mRNA vaccines, enabling rapid deployment during epidemics or pandemics. Several SANP-based vaccines are undergoing clinical trials [19,21,128].

Protein nanocages currently used for the development of novel vaccines include ferritin, lumazine synthase (LuS), Encapsulin, E2 protein, and small heat-shock proteins (sHSPs) (Figure 3). Each of these protein nanocages has distinct structural and immunological characteristics, allowing for the display of antigens on their surfaces through methods such as genetic fusion, tag coupling, or chemical conjugation to the N- and/or C-terminal regions, which are compatible with both prokaryotic and eukaryotic expression systems [129].

SAPNs-based vaccines utilize their unique nanoscale size (typically < 50 nm) and multivalent structure to mimic viral particles, enhancing immune activation. These nanoparticles are efficiently taken up by APCs and processed through the MHC-II pathway to activate CD4+ T cells, particularly Tfh cells. Simultaneously, their small size allows for direct interaction with naïve B cells, triggering germinal center reactions critical for high-affinity antibody production [19,128]. By synergizing BCR signaling with Tfh cell help, these antigens promote the survival, positive selection, clonal expansion, and affinity maturation of GC B cells, ensuring antibody diversity while preventing apoptosis through a balanced signaling interplay [130].

Previous studies reported neglectable anti-carrier antibodies to protein nanocages, indicating no adverse effects on vaccine immunogenicity [131]. Generally, these proteins alone are non-immunogenic, and those derived from bacterial sources do not induce autoimmunity [126,132]. Some of mentioned studies highlighted the role of adjuvants in the proper induction of immunity and in effectively engaging Tfh cells to SAPN vaccines (Table 1). SAPNs can also engage MHC-I pathways, stimulating CD8+ cytotoxic T cells, which provide cellular immunity by targeting infected cells [19,21]. This dual activation of humoral and cellular immunity is further supported by innate immune responses through PRR engagement. SAPNs mimic PAMPs to activate PRRs on DCs, triggering cytokine release (e.g., IL-21), antigen presentation, and Tfh cell differentiation [125].

SAPN vaccines effectively induce neutralizing antibodies and robust Tfh cell responses, which are critical for durable and cross-protective humoral immunity against rapidly mutating viruses. While Tfh cell responses have been extensively studied in humans and animal models, their evaluation in other species relies on indirect markers such as Tfh-associated cytokines or germinal center dynamics. Here, we describe examples of this multi-pathway mechanism of immune activation by protein nanocages.

Ferritin-based vaccines for chronic hepatitis B and SARS-CoV-2 have successfully induced efficient Tfh and GC responses [16,133]. Vaccination of mice with influenza HA–ferritin has also shown enhanced and longer-lasting GC responses in draining lymph nodes, focal deposition of the antigen in the GCs of these nodes, and increased BCR mutations in memory B cells following HA–ferritin immunization. However, no improvement in HA-specific Tfh cell responses was observed, suggesting that a highly structured and repetitive antigen array may promote germinal center formation through a B cell intrinsic mechanism independent of Tfh cell involvement [114]. IL-21 induction has been noted in response to the SARS-CoV-2 spike–ferritin vaccine, with the generation of long-lived plasma cells and the production of cross-neutralizing antibodies [10].

Similarly, lumazine synthase (LuS) and E2 nanoparticles, which are capable of presenting complex antigens, enhance Tfh cell activation by mimicking the repetitive antigenic patterns of natural pathogens, thereby facilitating optimal interactions between Tfh cells and antigen-specific B cells. However, studies using *Aquifex aeolicus* LuS have been limited to a few human pathogens, including HIV, SBV, SARS-CoV-2, and RVFV [21]. For example, a vaccine based on *A. aeolicus* LuS has been shown to protect approximately 80% of mice against a lethal dose of SBV and to produce virtually sterile immunity in cattle, even with a single dose of the vaccine [134]. The coupling of the SARS-CoV-2 spike protein with Lus has also been shown to elicit higher neutralizing responses than spike protein alone [135]. When multimerized with a rationally designed epitope (HIV-1 gp-120), LuS NPs have been found to activate both germline and mature VRC01 class B cells, which could be the basis for the broadly neutralizing antibody response to HIV-1 [136].

Recently, SARS-CoV-2 vaccine candidates were developed using an HR2-deleted glycine-capped spike (S2GΔHR2) displayed on ferritin and E2 protein. In mice, both SAPN vaccine candidates induced high neutralizing-antibody titers and robust T cell responses, including Th1, memory CD4+, CD107a-producing cytolytic CD4+ T cell, and GM-CSF–producing CD8+ effector T cell responses, highlighting their potential as vaccine candidates [137]. The 24-meric RBD–ferritin SAPN vaccines elicited stronger neutralizing-antibody responses compared to the RBD or stabilized spike (S2P) alone. However, S2GΔHR2 displayed on multilayered E2 proteins and I3-01v9 SAPNs achieved significantly higher titers, with an up to a 10-fold increase over S2P. While both ferritin and E2 protein platforms enhanced immunogenicity, the E2-based vaccine candidate showed a notable plateau in neutralizing-antibody responses after three doses, indicating the potential to reduce the dosage or number of injections without compromising efficacy [137]. This makes E2 protein an efficient platform for dose-sparing strategies, whereas ferritin provided robust baseline responses. E2 protein and LuS, with their 60-subunit structures, may have similar immunogenicity. However, compared to ferritin with 24 subunits, their larger size allows for the display of more antigen, potentially enhancing the immune responses and stability.

In this context, a subunit vaccine targeting zoonotic RVFV used the glycoprotein Gn head domain conjugated to LuS nanoparticles [116]. This vaccine candidate reduced mortality in a lethal mouse model and protected lambs, the most susceptible hosts, from viremia and clinical signs. Similarly, coupling the same subunit to E2 resulted in a vaccine candidate that was able to achieve full protection in lambs. However, to conclude that the type of SAPNs determines the immunogenicity, additional head-to-head comparisons are needed.

Encapsulin is another protein nanocage whose use in vaccines has shown effectiveness in inducing neutralizing antibodies against rotavirus, influenza, and COVID-19 (180-mer). Although Tfh cell responses were not explicitly evaluated, the vaccine candidate’s ability to stimulate robust humoral immunity was evident [115,138,139].

The potential of Encapsulin from Myxococcus xanthus as a scaffold for displaying 180 copies of the monomeric receptor-binding domain (mRBD) to enhance vaccine efficacy against SARS-CoV-2 variants in mice was evaluated [139]. The nanoparticle demonstrated long-term stability and, in mice, elicited high titers of neutralizing antibodies following a single immunization. A single booster enhanced these titers by 42-fold, effectively neutralizing the alpha, beta, and delta variants of concern (VOCs) with IC50 values comparable to the wild type. This platform proved highly effective in inducing robust neutralizing antibody responses. In mice, intramuscular immunization with oil-in-water adjuvanted formulations demonstrated that Encapsulin–pH1HA10 expressed in a prokaryote system provided complete protection against both homologous (Bel 09) and heterologous (PR8) influenza viral challenges [138].

In contrast, ferritin with the same antigen attachment and formulation offered partial protection and more pronounced clinical signs. This highlights *E. coli*’s suitability for generating simpler SAPNs and suggests that the number of subunits (180 in Encapsulin compared to 24 in ferritin) may play a critical role in enhancing antibody production and improving protective efficacy. The larger number of subunits in Encapsulin may contribute to more robust immune responses, underscoring the importance of nanoparticle design in vaccine effectiveness.

For rotavirus, the encapsulated (60-mer) vaccine candidate showed enhanced stability, and notable improvements in VP8*-specific IgG, including IgG1 and IgG2a, and neutralizing antibody responses with superior immunogenicity over a subunit vaccine in mice [115]. These responses indicate a balanced Th1/Th2 immune profile and capacity to promote cell-mediated immunity that is essential for effective viral vaccines.

Small heat shock proteins (sHSPs) hold promise as vaccine platforms, as they can promote durable antibody production and robust immune responses. Intranasal administration of an OVA–sHSP formulation in mice accelerated and enhanced OVA-specific IgG1 responses, with detection as early as 5 days post-immunization [18]. The study also revealed strong mucosal sIgA titers, germinal center accumulation of B and Tfh cells, and primed lung immunity, facilitating IgG and IgA responses upon influenza virus challenge. These findings underscore the potential of sHSPs to engage Tfh cells and support effective vaccine strategies, though further exploration is warranted.

Considering the numerous advantages of SAPN platforms, such as their ability to create safe, effective, and stable vaccines suitable for mucosal immunization and oral delivery, it is imperative to evaluate and optimize the various aspects of their application. Specifically, strategies like the SpyTag/SpyCatcher system or protein-induced nanocage (PINC) technology [140] for antigen attachment and adjuvant encapsulation targeting Tfh cell activation, such as saponins and TLR agonists, could significantly enhance vaccine efficacy. Delivering adjuvants with nanocage vaccine candidates shows promise for engaging Tfh responses, promoting durable antibody production and providing cross-protection against emerging viral strains. The recent use of SAPNs in mRNA vaccines further highlights their significant role and potential in vaccinology.

### 4.4. Self-Assembling Protein Nanocages in RNA Vaccine Design (SAPN-RNA Vaccines)

Most viruses, including influenza, undergo rapid antigenic changes through drift and shift. Therefore, the ultimate goal is to develop a universal vaccine that offers broad protection against diverse strains and emerging pandemic threats [141].

The incorporation of SAPNs with the mRNA approach (SAPN-RNA vaccines) represents a synergistic strategy to enhance vaccine efficacy with cross-protection potential against different viral strains. SAPNs, with their multivalent antigen display and nanoscale architecture, further amplify immune responses by directly engaging B cells and facilitating germinal center formation, as well as activation of PRRs, while also reinforcing the LNP formulation for improved uptake and more efficient antigen delivery [19,128]. Based on recent studies, this approach can induce robust Tfh-mediated antibody responses at lower doses while enhancing stability, versatility, and cross-protection against evolving pathogens (Table 1). Additionally, SAPNs may extend the immune response duration of mRNA vaccines. Studies on ferritin-fused mRNA vaccines for HIV and SARS-CoV-2 demonstrated significant improvements in immune responses compared to traditional mRNA or subunit vaccines [88,111]. Ferritin-fused mRNA vaccines induced stronger Tfh cell activation and enhanced GC responses, leading to higher frequencies of GC Tfh cells and elevated IL-21 cytokine levels [88,111]. An mRNA-encoded HIV-1 Env trimer ferritin nanoparticle vaccine demonstrated an ability to elicit robust GC B cell, memory B cell, and Tfh cell responses in mice [88].

Incorporating LuS self-assembling protein nanoparticles into mRNA vaccines enhances immunogenicity by forming 60-mer nanoparticles that mimic viral structures. For SARS-CoV-2, an mRNA vaccine using LuS nanoparticles to display multivalent RBD Delta induced strong neutralizing antibody responses and provided protection against the Delta variant in mice. The platform also allows for the design of a bivalent vaccine encoding wild-type and alpha variants, demonstrating comparable antibody responses to the monovalent version, highlighting its versatility [113].

An ongoing Phase 1 clinical trial (NCT05001373) evaluating the safety and immunogenicity of LuS-based HIV mRNA vaccines is expected to provide further insights into the LuS-specific immune responses elicited in humans after repeated vaccinations. Broadly neutralizing HIV antibodies typically require high levels of somatic hypermutation and specialized germline features, presenting challenges for vaccine development. To address this, an LNP-vectored alphavirus replicon encoding the GT eOD-GT8 immunogen, fused to LuS, was used to enhance the development of broadly neutralizing responses against HIV [22]. In the clinical trial, replicon immunization induced a slight, though not statistically significant, increase in the total number of CD4+CXCR5+ Tfh cells compared to protein-based vaccines. GC Tfh cells were induced at similar levels in both groups, but replicon immunization resulted in higher upregulation of the ICOS costimulatory receptor on Tfh cells early in the immune response, indicating that replicon vaccines may induce equivalent or superior GC responses, enhancing the potential for broadly neutralizing HIV antibodies [22]. Peak Tfh responses were also observed by day 12, with a slow decay thereafter, suggesting that the incorporation of SAPNs such as Lus may help facilitate slower antigen release and extend the duration of the immune response

LuS nanocages have also been employed in the context of RNA replicon vaccines against rotavirus [112]. LuS mRNA vaccines encoding VP8* self-assemble into 60-mer nanoparticles that mimic viral structures, effectively enhancing antigen presentation. These vaccines demonstrated robust immunogenicity in rodents, eliciting both humoral and cellular immune responses. In guinea pigs, LuS-P2-VP8* consistently induced higher antibody levels than P2-VP8* mRNA over a six-month period in both monovalent and trivalent formulations targeting P8], P6], and P4] genotypes. Moreover, the mRNA vaccines generated superior virus-neutralizing antibody responses and VP8*-specific T cell responses, which were absent in the alum-adjuvanted protein vaccine groups [112]. However, despite the high levels of neutralizing antibodies, the role of Tfh cells in LuS-based mRNA vaccines remains unexplored.

### 4.5. Adjuvants Promote Tfh Cell Differentiation and Responses

Adjuvants are essential component of non-live vaccines as they enhance immunogenicity by activating innate immune pathways, promote GC reactions, and drive the differentiation of Tfh cells. Certain delivery materials can also act as adjuvants by mimicking the size or structure of natural pathogens, facilitating antigen uptake and presentation by APCs through PRR signaling [11]. The selection and design of adjuvants directly influence the magnitude and quality of Tfh responses, which are especially important for targeting viral pathogens [10,142].

Aluminum salts (alum), one of the most extensively used vaccine adjuvants, primarily induce the development of Th2 and Tfh cells, promoting the production and secretion of high-affinity antibodies [143]. Demonstrating superior efficacy over MF59 in certain contexts, alum enhances early germinal center formation in response to peptide–protein conjugates and improves vaccine efficacy against non-viral disorders [144]. Alum adjuvants have also been used in SAPN vaccines to engage Tfh and GC responses (Table 1). Nonetheless, concerns about the local side effects of this adjuvant, along with its limited ability to elicit robust Tfh responses, highlight the necessity for more advanced adjuvant systems to address the challenges posed by viruses [145].

Recent advancement in adjuvant technology have introduced formulations targeting immune pathways to enhance Tfh cell responses. Oil-in-water emulsions like MF59 promote Tfh differentiation via IL-6-driven expression of Bcl6, a key transcription factor, and have demonstrated success in human influenza vaccines by boosting germinal center reactions and antibody responses [146]. Montanide™ ISA 206 is another example that has shown efficacy in veterinary vaccines, such as those for PRRSV, by enhancing germinal center formation and antibody production [147]. In the context of SAPN vaccines for viruses, oil-in-water emulsions such as MF59 have been used in ferritin-based SARS-CoV-2 and HIV vaccines, as well as in Encapsulin-based IAV and EBV vaccines, while Montanide ISA201VG was used to support FMDV–ferritin vaccines [21].

Toll-like receptor (TLR) agonists are a class of adjuvants that have demonstrated significant promise in enhancing Tfh and antibody responses [148,149]. TLR4 agonists, such as monophosphoryl lipid A (MPLA), and TLR9 agonists, like CpG oligodeoxynucleotides, are more effective than alum at stimulating immune responses by activating the innate immune system using potent danger signals [11].

Moreover, TLR9 and STING agonists have been shown to synergistically enhance the SARS-CoV-2 RBD vaccine response by boosting GC B cells and modulating Tfh responses. The combination of CpG-2722 and STING ligands elicited robust GC Tfh (CD4+Bcl-6+CXCR5+ICOS+) and B cell responses in draining lymph nodes and spleens, demonstrating their cooperative potential to improve vaccine efficacy [16].

TLR7/8 agonists are another promising vaccine adjuvant due to their ability to activate APCs and enhance Th1 and Tfh responses, which are critical for viral immunity. In a non-human primate model, TLR7 adjuvantation induced robust HIV Env-specific CD4+ T cell responses, and enhanced Tfh differentiation and germinal center formation [148], while TLR8 is a key sensor of vita-PAMPs and driver of Tfh differentiation; thus, TLR7/8 agonists have potential in Tfh-skewing vaccine adjuvant strategies [149].

MPLA enhances the immune response to inactivated rabies vaccines by promoting the generation of Tfh cells, GC B cells, and plasma cells, which increases the production of RABV-specific IgG, IgG2a, IgG2b, and virus-neutralizing antibodies, indicating its potential in improving immune responses against zoonotic viruses [150]. Notably, MPLA, a key component of the adjuvants AS01 (containing QS-21, a saponin-based adjuvant) and AS04 (containing aluminum salts), has been successfully used in licensed vaccines, including AS01 for malaria [151] and AS04 for HPV [152]. AS04 induces a Th1-biased immune response, while AS01, which also enhances Tfh cell responses, is being evaluated in vaccines targeting viral infections [153]. Saponin/MPLA nanoparticles showed greater potency than AS01B in mice and primed robust immune responses, including germinal center B cell, Tfh cell, and HIV tier 2 neutralizing antibody responses in non-human primates [154].

NKT cell-based adjuvants are an effective strategy for enhancing vaccine efficacy by promoting early cytokine production, which supports Tfh cell differentiation and B cell activation, thereby boosting humoral immunity [155]. These cells are activated by lipid antigens presented by CD1d molecules, secreting cytokines like IL-4, IL-21, and IFN-γ that strengthen the adaptive immune response [40,41]. An example is α-galactosylceramide (α-GalCer), a glycolipid that activates invariant NKT cells. In murine studies, co-administration of α-GalCer with an influenza virus vaccine enhanced germinal center reactions, leading to the production of high-affinity antibodies against the virus [156].

Another approach involves adjuvants that target mucosa-associated invariant T (MAIT) cells. There is evidence that intranasal immunization with MAIT cell agonists, combined with protein antigens like the spike RBD of SARS-CoV-2 and influenza hemagglutinin, induces protective humoral immunity and IgA production. The MAIT cell-mediated adjuvant activity is driven by CD40L-dependent dendritic cell activation and Tfh cell priming, with antibody responses comparable to those induced by the NKT cell agonist α-GalCer [157].

Comparative studies have underscored the importance of adjuvant selection in shaping Tfh responses. For instance, a malaria P27A peptide vaccine formulated with the experimental adjuvant GLA-SE elicited significantly higher circulating Tfh activation and antibody titers than alum-based formulations [145]. Similarly, the PfRH5 antigen combined with AS01 produced stronger Tfh responses compared to a heterologous viral vector prime-boost regimen [153].

Nanoencapsulation can enhance the efficacy of classical vaccines by inducing CD4+ Th17 and Tfh cell responses, which are crucial for promoting robust humoral immunity. This mechanism contributes to improved vaccine outcomes by facilitating antigen presentation and promoting germinal center formation [75].

Despite the self-adjuvant role of LNPs, some studies explored combining mRNA vaccines with traditional adjuvants, like alum, to further enhance immune activation [93,112]. mRNA vaccines encoding the HIV-1 Env trimer, in combination with ferritin NPs, induced monoclonal antibodies that neutralized diverse HIV-1 isolates, further demonstrating the potential of mRNA-LNP and SAPN platforms to stimulate potent Tfh and GC responses [88].

While SAPN vaccines enhance neutralizing-antibody production, adjuvants can help achieve complete protection by optimizing immune responses [21]. As demonstrated with the ALFQ formulation, combining MPLA and QS-21 significantly boosted antigen-specific immune responses in SARS-CoV-2–ferritin vaccine models, leading to potent polyfunctional cytokine responses and long-lived memory CD8+ T cells [10]. This lipid formulation or the different dosing regimen of this unique adjuvant can extend the duration of antigen presentation.

In chronic hepatitis B (HBV), the combination of ferritin nanoparticles and CpG-1826 enhanced Tfh cell and germinal center B cell responses, improving antibody production [15]. Similarly, squalene-based formulations like AddaVax™, similar to MF59, have been shown to drive extended germinal center activity and memory B cell maturation in influenza ferritin-based vaccines [114]. These adjuvants promote cytokine expression, Tfh cell recruitment, and B cell activation, aiding immune memory formation and effective antigen targeting.

Future directions in adjuvant research should focus on refining Tfh responses by modulating cytokine pathways such as IL-6 and IL-27 signaling or suppressing IL-2, which are critical for Tfh differentiation and germinal center maintenance. Innovative adjuvants like QS-21 saponins, STING agonists, and nanoparticles provide new strategies for optimizing immune responses and vaccine design. Future approaches may also incorporate IL-21 as an immune adjuvant to enhance humoral immunity and promote Tfh activation. Additionally, probiotics, whether as non-replicating vectors [158] or vaccination adjuncts, could further amplify Tfh-mediated immunity [32], particularly in mRNA and SAPN-based vaccines. These advancements in adjuvants and vaccine design hold promise for next-generation vaccines that elicit durable, high-quality immunity.

## 5. Challenges and Future Directions

Tfh cells are essential for generating long-lasting humoral immunity, driving B cell maturation, germinal center formation, and high-affinity antibody production, making them crucial for immune responses against viral infections. Their central role has positioned them as key targets in vaccine development aimed at enhancing antibody responses. However, several challenges remain in fully utilizing Tfh cells for immunotherapy and vaccine design.

A major challenge in evaluating Tfh cells during viral infections is their transient nature and specialized localization in secondary lymphoid tissues like lymph nodes, making them difficult to study in vivo. Their activation is regulated by a complex network of cytokines and interactions with B cells, which are context-dependent and often short-lived, complicating the ability to track and analyze Tfh cell responses during an active viral infection.

Chronic viral infections impair Tfh cell function, disrupting B cell support and leading to suboptimal antibody responses. This dysfunction, along with immune dysregulation, complicates Tfh evaluation. Identifying antigen-specific Tfh cells is challenging due to their low frequency and transient expression of markers like PD-1 and CXCR5. Advancing techniques such as single-cell RNA sequencing and spatial transcriptomics, along with developing species-specific antibodies targeting CD4+ and GC Tfh cells, will improve our understanding of Tfh cells’ roles in viral infections and support vaccine development.

Circulating Tfh (cTfh) cells are key biomarkers of systemic immune responses, especially in vaccine-induced immunity. While mainly studied in humans, their heterogeneity, unclear relationship with tissue-resident Tfh cells, and dynamic fluctuations complicate their use as reliable correlates of protection. Future research should prioritize refining cTfh phenotyping, standardizing measurement protocols, and exploring their role across diverse infectious diseases and vaccine platforms, which could also enhance viral control and vaccine efficacy in veterinary species exhibiting species-specific immune variations.

Exploring vaccines that induce Tfh cell responses is critical for enhancing protective immunity against viral infections. The development of novel vaccine platforms, such as mRNA vaccines and self-assembling protein nanocages, as well as their integration into synergistic strategies like SAPN-RNA vaccines, offers promising solutions to stimulate Tfh activation and improve antibody responses. Additionally, the inclusion of targeted adjuvants may further optimize these vaccines for stronger and longer-lasting immunity. Adjuvants such as TLR agonists, saponins, and STING agonists, along with another strategy like dendritic cell mRNA vaccines, may further support post-vaccination Tfh cell function during viral infections.

In summary, the development of vaccines that engage Tfh cell responses offers significant potential to improve prevention and control strategies for viral infections in both human and veterinary medicine.

## Data Availability

No new data were created or analyzed in this study. Data sharing is not applicable to this article.
